# Applications of CRISPR/Cas13-Based RNA Editing in Plants

**DOI:** 10.3390/cells11172665

**Published:** 2022-08-27

**Authors:** Naga Rajitha Kavuri, Manikandan Ramasamy, Yiping Qi, Kranthi Mandadi

**Affiliations:** 1 Texas A&M AgriLife Research and Extension Center, Texas A&M University System, 2415 E. Highway 83, Weslaco, TX 78596, USA; 2 Department of Plant Pathology and Microbiology, Texas A&M University System, 2132 TAMU, College Station, TX 77843, USA; 3 Department of Plant Science and Landscape Architecture, University of Maryland, College Park, MD 20742, USA; 4 Institute for Bioscience and Biotechnology Research, University of Maryland, Rockville, MD 20850, USA; 5 Institute for Advancing Health Through Agriculture, Texas A&M AgriLife, College Station, TX 77845, USA

**Keywords:** type VI CRISPR systems, CRISPR/Cas13, RNA targeting, RNA editing, RNA interference

## Abstract

The Clustered Regularly Interspaced Short Palindromic Repeats (CRISPR)/CRISPR-associated (Cas) system is widely used as a genome-editing tool in various organisms, including plants, to elucidate the fundamental understanding of gene function, disease diagnostics, and crop improvement. Among the CRISPR/Cas systems, Cas9 is one of the widely used nucleases for DNA modifications, but manipulation of RNA at the post-transcriptional level is limited. The recently identified type VI CRISPR/Cas systems provide a platform for precise RNA manipulation without permanent changes to the genome. Several studies reported efficient application of Cas13 in RNA studies, such as viral interference, RNA knockdown, and RNA detection in various organisms. Cas13 was also used to produce virus resistance in plants, as most plant viruses are RNA viruses. However, the application of CRISPR/Cas13 to studies of plant RNA biology is still in its infancy. This review discusses the current and prospective applications of CRISPR/Cas13-based RNA editing technologies in plants.

## 1. CRISPR/Cas Systems

Clustered Regularly Interspaced Short Palindromic Repeats (CRISPR) systems are found in prokaryotes such as archaea (~80%) and bacteria (~40%) [1]. CRISPR consists of identical direct repetitive DNA sequences (direct repeats), interspaced by highly variable sequence (spacer), and are often associated with CRISPR-associated (Cas) proteins [1,2]. CRISPR, along with the Cas protein, provides an adaptive immune system in prokaryotes targeting the genetic material of the invading pathogen [2]. The discovery of this mechanism in prokaryotes has driven scientists to adopt the CRISPR/Cas system as an advanced tool to modify DNA and RNA in various organisms. CRISPR/Cas systems are classified mainly into class 1, where a multi-protein effector complex is present, and class 2, which utilizes a single protein to edit the target [3]. These classes are further divided into types and sub-types based on the variations and functions of Cas proteins [4,5].

Class 1 Cas systems comprise types I, III, and IV with diverse variants and effector complexes, whereas class 2 Cas systems consist of types II, V, and VI with an effector module containing a single multifunctional protein [5]. A typical CRISPR/Cas genome editing tool consists of a guide RNA (gRNA or CRISPR RNA (crRNA)) complementary to the gene target site and a Cas effector protein, which acts as an endonuclease to cleave the target site [6]. Because of their more straightforward organization, class 2 Cas systems are most widely employed as genome editing tools in which type II and V systems use Cas9 and Cas12 enzymes to edit DNA. Notably, the class 2 CRISPR/Cas9 protein of type II is one of the first Cas proteins studied in detail, which led to its widespread application for DNA editing in animals, plants, and bacteria [7,8,9,10,11]. However, there are certain limitations in DNA editing using CRISPR, such as the requirement of a Protospacer Adjacent Motif (PAM) site, off-target mutations, and low efficiency against viruses [12,13]. Some of these limitations can be circumvented by the recently identified type VI CRISPR/Cas systems that use the Cas13 protein to enable sequence-specific cleavage of ssRNA molecules [14]. RNA manipulation is advantageous over DNA editing as it prevents unwanted pleiotropic effects, and RNA products can be precisely and spatiotemporally regulated [15]. Here, we discuss the recent advances in CRISPR/Cas13 systems for RNA editing, focusing on their applications in plants and future directions.

## 2. Overview of CRISPR/Cas13 Systems

A single multifunctional Cas13 effector protein contains two higher eukaryotes and prokaryotes nucleotide-binding domains (HEPN) that provide RNase activity. The Cas13 protein, when associated with crRNA, forms an RNA-guided RNA targeting complex to recognize and cleave ssRNA targets. Based on Cas13 phylogeny, features, and functional characterization, this system is further classified into six subtypes: VI-A (Cas13a, C2c2), VI-B (Cas13b, C2c4), VI-C (Cas13c, C2c7), VI-D (Cas13d), and recently, VI-X (Cas13X) and VI-Y (Cas13Y) [16,17,18,19,20] (Table 1). All Cas13 proteins possess two enzymatically distinct RNase activities, which include processing pre-crRNA into mature functional crRNA and the degradation of target RNA by the HEPN domains [17,21,22,23]. The location of these HEPN domains differs based on the type of Cas13 proteins. In Cas13a, 13c, and 13d, the HEPN domains are present at the center and C terminus [24], whereas in Cas13b, Cas13X, and Y, they are located at the N-terminus and C-terminus of the proteins [17,20]. The HEPN domains of Cas13 proteins can cleave not only the desired target, but also exhibit a non-specific collateral cleavage activity resulting in the degradation of the RNA near the Cas13 complex [17,18,24]. The length of the crRNA or the spacer sequence varies (24–30 nt) with the type of Cas13. Of all the Cas13 enzymes, Cas13c is the least functionally characterized. This section discusses different subtypes of the type VI CRISPR/Cas system.

### CRISPR/Cas13 Classification

Cas13a, or type VI-A, is the first subtype identified among the type VI systems and was characterized in *Leptotrichia shahii* (Lsh) [16,24]. In addition, Cas13a has many orthologs such as *Listeria seeligeri* (Lse) and *Leptotrichia wadei* (Lwa) [25], *Leptotrichia buccalis* (Lbu) [21], and *Lachnospiraceae bacterium* (Lba) [26] (Table 1). The Cas13a CRISPR array typically consists of a 5′ 28 nt direct repeat (DR) unique to each ortholog and a 28-30 nt spacer sequence (complementary to the target sequence). Some orthologs such as LshCas13a have a 3′ H (non-G), a single base protospacer flanking site (PFS) preference, whereas LwaCas13a and LbuCas13a do not show any PFS preference.

Cas13b has its DR on the 3′ end of crRNA compared to the 5′ DR present in Cas13a, Cas13c, and Cas13d. Cas13b orthologs such as *Bergeyella zoohelcum* (BzCas13b) and Porphyromonas gulae (PguCas13b) prefer 5′ PFS of D (A, U, or G) and 3′ PFS of NAN or NNA (Table 1) [17]. However, Cas13b from *Prevotella* sp. (PspCas13b) has no PFS requirement [27]. Cas13b is further differentiated into two types based on the presence of regulatory accessory proteins csx27 and csx28 that can repress or enhance the RNA interference activity of Cas13b, respectively [17].

Cas13c is not as functionally characterized compared to other types of Cas13. However, the known characteristics, such as the presence of DR on the 5′ end of crRNA and the spacer length (28–30 nt), are similar to that of Cas13a and Cas13d (Table 1) [19,28]. Cas13c is less efficient at RNA targeting when compared to the Cas13a, b, and d efficiencies [28]. Therefore, most current Cas13 studies rarely employ Cas13c orthologs such as *Fusobacterium perfoetens* (FpeCas13c).

Cas13d is the smallest of Cas13a-d and is one of the most efficient type VI systems for RNA targeting [18,22] (Table 1). The Cas13d from the *Ruminococcus flavefaciens* XPD3002 (CasRx/RfxCas13d) is a prominent homolog for multiple organisms [29,30,31]. In addition to the Cas13d effector protein, type VI-D contains a WYL domain consisting of accessory proteins, one of which can positively modulate target and collateral RNase activity [18]. Type VI-D is known for its versatility as it has no PFS constraints like the other Cas13 enzymes.

**Table 1:** Summary table of CRISPR/Cas13 classification.

Type of Cas13	Orthologues	Structural Composition	Functional Region	Application Scope	Reference
Cas13a (1250 aa)	LshCas13a	HEPN Domains (center and C terminus), pre-CrRNA processing, 3′ Non-G PFS preference (except Lwa and LbuCas13a), DR present on the 5′ end	ssRNA (spacer length 28–30 nt)	Virus resistance, RNA knockdown, disease diagnostics	[21,24,26,32]
	LseCas13a				
	LwaCas13a				
	LbuCas13a				
	LbaCas13a				
Cas13b (1150 aa)	BzCas13b	HEPN Domains (N and C terminus), pre-CrRNA processing, 5′ PFS preference of D, 3′ PFS NAN/NNA (Except PspCas13b), DR present on 3′ end	ssRNA (spacer length 30 nt)	Virus resistance, RNA base editing, RNA knockdown	[17,33]
	PguCas13b				
	PspCas13b				
Cas13c (1120 aa)	FpeCas13c	HEPN domains (center and C terminus), pre-CrRNA processing, No PFS preference, DR present on 5′ end	ssRNA (spacer length 28–30 nt)	RNA knockdown	[19,28]
Cas13d (930 aa)	RfxCas13d	HEPN domains (center and C terminus), pre-CrRNA processing, No PFS preference, DR present on 5′ end	ssRNA (spacer length 23–30 nt)	Virus resistance, RNA knockdown, alternative splicing modulation	[34,35]

## 3. Applications of CRISPR/Cas13 Systems in Plants

Type VI CRISPR/Cas systems provide various applications in various organisms through different RNA technologies such as RNA interference, RNA detection, RNA editing, and RNA targeting [36,37,38]. Cas13 has been used in plants to target RNA viruses, with very few studies on endogenous plant RNA. However, the recent applications of Cas13 in transcriptome studies of humans, animals, and pathogens are opening possibilities for its application in plants. Here, we discuss different CRISPR/Cas13-based RNA technologies and their current plant applications.

### 3.1. RNA Interference against Viruses

**Figure 1:**
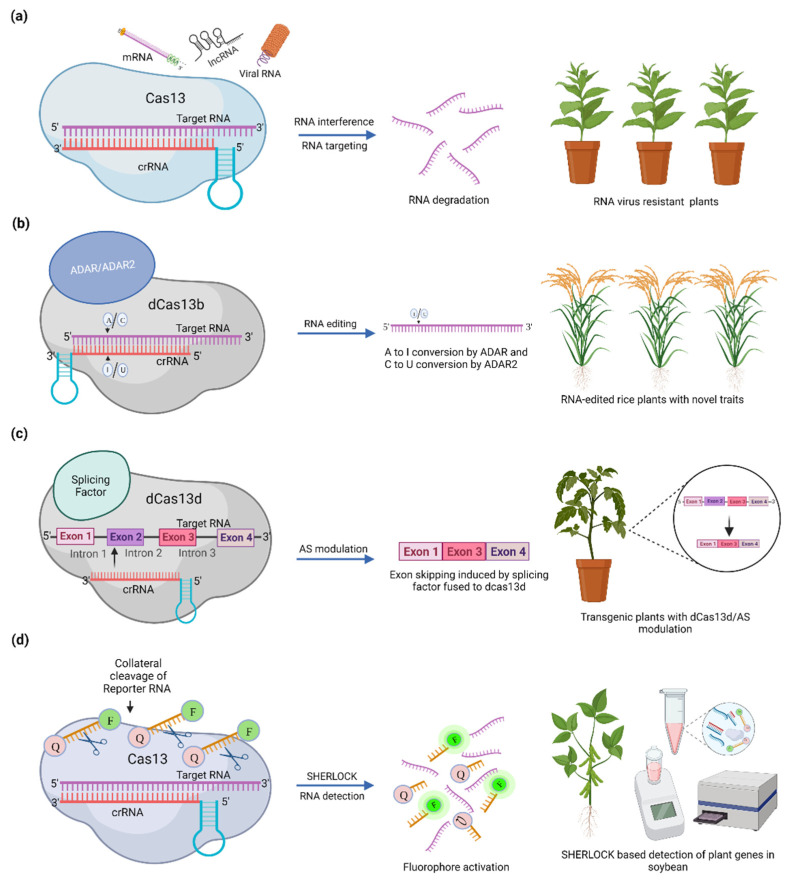
Pictorial representation of CRISPR/Cas13-based RNA technologies. (**a**) RNA interference of virus and RNA targeting using Cas13 to generate virus-resistant plants. (**b**) RNA editing illustration of A to I and C to U conversions when dCas13b is fused with ADAR or ADAR2 enzymes to manipulate a gene’s function and generate transgenic plants with novel traits. (**c**) Alternative splicing (AS) modulation with splicing factor fused to dCas13d to elucidate the function of different alternatively spliced transcripts. (**d**) RNA detection using Cas13-based SHERLOCK method for rapid detection of plant genes and pathogens.

RNA interference (RNAi) is an innate antiviral immunity mechanism that has been successfully used to combat various plant viruses. Nevertheless, the availability of such antiviral strategies is still limited to specific virus groups. Many viruses readily mutate and have developed different counter-defense mechanisms, leading to the rapid emergence of new antiviral approaches. The recently developed CRISPR/Cas13 is a promising tool for engineering plant immunity against a broad range of RNA viruses that constitute a majority of plant viruses (Figure 1a). Although similar to RNAi technology, Cas13 can be highly specific, resulting in fewer off-targets and high knockdown efficiency [28,39]. Aman et al. have successfully demonstrated viral RNA interference using LshCas13a against the *Turnip Mosaic Virus* (TuMV) RNA genome, targeting its helper component proteinase (HC-Pro) and coat protein (CP) sequences in *Nicotiana benthamiana* and *Arabidopsis thaliana,* respectively [40,41] (Table 2). Subsequently, CRISPR/Cas13 has been applied to target RNA viruses such as the *potato virus Y* (PVY), *tobacco mosaic virus* (TMV), *southern rice black-streaked dwarf virus* (SRBSDV), and *rice stripe mosaic virus* (RSMV) in various plants [42,43] (Table 2). Mahas et al. [30] have demonstrated that CasRx/Cas13d mediates high interference compared to Cas13a and Cas13b simultaneously against one or two different RNA viruses. A recent study on Sweet Potato Virus Disease (SPVD) resistance using the CRISPR/Cas13 system demonstrated that LwaCas13a and RfxCas13d exhibited efficient targeting activity against multiple RNA viruses simultaneously [44].

### 3.2. RNA Targeting/Knockdown

The CRISPR/Cas13 system can target any single-stranded RNA for degradation without altering DNA (Figure 1a). Accordingly, in addition to RNA interference against viruses, several Cas13 variants have been successfully applied to target endogenous RNA transcripts of various organisms, including LwaCas13a with efficient mRNA knockdown in human cancer cells, RfxCas13d in mRNA knockdown of zebrafish embryos [45], porcine cell, and parthenogenetic embryos [31]. In plants, mRNA knockdown using LwaCas13a was successfully performed in rice protoplasts targeting three different endogenous transcripts, such as 5-enolpyruvylshikimate-3-phosphate synthase (EPSPS), hydroxycinnamoyl transferase (HCT), and phytoene desaturase (PDS), resulting in more than 50% knockdown efficiency in 48 h [25] (Table 2). Recently, Sharma et al. demonstrated the knockdown of PDS transcript in *N. benthamiana*, *A. thaliana*, and *Solanum lycopersicum* using LbaCas13a and LbuCas13a via *Agrobacterium* infiltration. This study also found that the crRNA can induce gene silencing even in the absence of Cas by utilizing the Argonaute proteins and the plant RNAi machinery [15]. Apart from mRNA, functional studies targeting different non-coding RNA such as circular RNA (circRNA) [46,47] and long non-coding RNA (lncRNA) [48,49] were demonstrated in several human cancer studies. In plants, non-coding RNA knockdown using CRISPR/Cas13 systems has not yet been performed and could be a potential application to characterize the roles and relations of lncRNA with protein-coding mRNA in plants.

### 3.3. RNA Editing

RNA editing involves the post-transcriptional editing of RNA in a site-specific manner. Recently, Cox et al. engineered a deactivated Cas13b (dCas13b) that binds to target RNA but lacks endonuclease activity [27], allowing the authors to associate the dCas13b enzyme with the ADAR (Adenosine Deaminase Acting on RNA) family enzyme that provides Adenosine to Inosine (A to I) editing of double-stranded RNA (dsRNA). This system can be further adapted to base edit the endogenous transcripts, increase the diversity of the transcriptome, and determine its significance. Based on this concept, RNA editing tools such as RNA Editing for Programmable Adenosine to Inosine (A to I) Replacement (REPAIR) [27] and RNA Editing for Specific Cytosine to Uracil (C to U) Exchange (RESCUE) [50] have been engineered and successfully applied in insects and mammals [20,27,28,50,51]. The ADAR2 deaminase domain provides the base editing from A to I (REPAIR) and C to U (with RNA cytosine deaminase from evolved ADAR2) (RESCUE) without cleaving the RNA transcript (Figure 1b). The A to I and C to U editing of RNA can provide mRNA manipulation, causing alterations in splicing and translation without making permanent changes at the genomic level. Base editing of C to T and A to G of the nuclear genome was previously demonstrated in plants using cytidine base editors (CBEs) and adenine base editors (ABEs), respectively, using a Cas9 nickase [52,53,54,55,56]. These techniques open doors for CRISPR/Cas13 base editors for potential editing in plants at the RNA level.

### 3.4. Modulation of Alternative Splicing

Alternative splicing is a process regulating gene expression in which exons of a gene are spliced to form multiple alternative transcripts. The CRISPR/Cas13 system can regulate RNA splicing pathways by introducing point mutations into the 5′ and 3′ ends of a splice donor site (GU) and a splice acceptor site (AG), respectively, resulting in mis-splicing and losing targeted splice variants. Konerrman et al. [22] demonstrated a proof of concept for the manipulation of alternative splicing by fusing a catalytically inactive CasRx (dCasRx) with a Gly-rich C-terminal domain of a heterogeneous nuclear ribonucleoprotein (hnRNPa1) splice factor to induce exon skipping in Microtubule Associated Protein Tau (MAPT) pre-mRNA that encodes tau proteins in human neurodegeneration. Recently, Cas13-based splicing modulation tools such as CRISPR Artificial splicing factors (CASFx) have been engineered to demonstrate simultaneous exon inclusion and exclusion induced in different RNA targets based on the positioning of CASFx [35] (Figure 1c). In plants, the potential use of CRISPR/Cas13 to modulate alternative splicing in serine/arginine-rich (SR) family proteins has been described to analyze the role of splice isoforms in stress responses [57]. Nevertheless, experimental studies on CRISPR/Cas13 to manipulate alternative splicing in plants are yet to be demonstrated. The studies mentioned above provide feasibility for potentially modulating alternative splicing in plant disease-susceptible genes to achieve crop improvement.

### 3.5. RNA Tracking and Nucleic Acid Detection

The target RNA recognition and binding abilities of CRISPR/Cas13 provide means for its application in various functional and diagnostic studies. Previously, Abudayyeh et al. [25] harnessed the catalytically inactive LwaCas13a (dLwaCas13a) to bind targeted RNA transcripts and track stress granule formation in mammalian cells. Later, Yang et al. [58] performed dynamic RNA imaging in live cells using different CRISPR/Cas13 systems. RNA tracking using Cas13 has not been implemented in plants yet. However, in addition to RNA tracking, Cas13 is becoming a prominent tool in diagnostics for nucleic acid detection focusing on viral diseases [33,59]. The targeted cleavage of ssRNA by Cas13 is followed by a non-specific collateral cleavage of proximal RNA. East-Seletsky et al. [21] leveraged this collateral activity of Cas13 to develop an RNA detection tool that can be utilized in vitro to collaterally cleave a fluorophore quencher-labeled reporter RNA that can result in increased fluorescence upon target-RNA-triggered RNase activation (Figure 1d). The Cas13-based RNA detection tool Specific High sensitivity Enzyme Reporter unlocking (SHERLOCK) [60] was further developed based on this concept (Figure 1d). Recently, Ackerman et al. [61] developed a high throughput tool that uses Combinatorial Arrayed Reactions for Multiplexed Evaluation of Nucleic acids (CARMEN). CARMEN, combined with Cas13, can simultaneously detect 4500 crRNA target pairs on a single array, thereby providing a gateway for multiplexed pathogen detection using CRISPR/Cas13. Nucleic acid detection of plant genes using CRISPR/Cas13 was demonstrated by Abudayyeh et al. [32] in soybeans to detect multiple genes in a single reaction using SHERLOCK and to quantify levels of a glyphosate resistance gene, EPSPS from *Agrobacterium* sp. strain CP4 (CP4 EPSPS), in a mixture of soybeans (Table 2). This platform could be used in other agricultural contexts, such as detecting and quantifying genes, and can also be leveraged for the early detection of plant pathogens or pests, enabling rapid responses by farmers to reduce the use of pesticides or herbicides.

## 4. Prospective Directions of CRISPR/Cas13 RNA Targeting Systems in Plants

CRISPR/Cas technology has become a powerful genome-editing tool due to its applications in various fields for genetic alterations, disease diagnostics, and crop improvement. For the past decade, the DNA targeting CRISPR/Cas9 system has evolved into a remarkable tool for its implementation in various DNA technologies such as gene knockout, gene activation, gene editing, gene therapy, and DNA detection in multiple organisms [62,63,64,65,66,67]. The recent discovery of CRISPR/Cas13 RNA targeting systems aids in the advancement of existing RNA technologies and has become a promising tool applicable to various organisms [28,45,48]. However, the recent studies on CRISPR/Cas13 systems in plants have mainly focused on viral RNA interference. Cas13-mediated knockdown of endogenous mRNA has been demonstrated in a few studies [15,25], targeting known genes as a proof of concept that can be applied to target the mRNA of disease-susceptible plant genes or study the function of unknown genes through transient knockdown without disrupting the DNA sequence.

In addition, targeting non-coding RNA such as long non-coding RNA, microRNA, and circular RNA in plants using CRISPR/Cas13 can become a promising tool to study their role in plants. Previously, targeting non-coding RNA in plants was performed using CRISPR/Cas9 at the DNA level to find their roles in plant growth, development, and stress responses [52,68,69,70,71,72]. Similarly, CRISPR/Cas13 can also be employed to target non-coding RNA in plants at the RNA level, thereby promoting plant resistance without altering the genome. In the recent past, base editing in plants was performed using an impaired Cas9 (nCas9 or dCas9) to make single base alterations (C to T or T to A) in the genome [52,71,72]. Similarly, RNA base editing can be performed through REPAIR and RESCUE tools that use dCas13 fused with deaminase enzymes causing A to G and C to U conversions [27,73]. However, questions were raised addressing the challenges in the expression stability of CRISPR base editors and in identifying the phenotypic variations caused by base changes in RNA transcript in recent reviews on base editing using CRISPR/Cas13 in plants [74,75]. Therefore, more research on RNA editing in plants using CRISPR/Cas13 is needed for its application in future crop improvement. [74,75].

## 5. Potential Limitations of CRISPR/Cas13

Though CRISPR/Cas13 can be used to target ssRNA in many organisms, the RNA targeting and RNA binding in Cas13 have constraints, some of which might limit its use. One major limitation is the collateral activity of Cas13 that causes non-specific RNA cleavage [76,77,78]. To overcome this limitation, Tong et al. [79] recently developed high-fidelity Cas13 variants with a dual-fluorescent-reporter system to detect and screen Cas13 variants in mammalian cells, providing efficient RNA targeting and minimizing collateral effects. However, no such collateral activity has been observed in plants yet. Apart from this, few studies in insects and plants report the knockdown of target RNA by crRNA alone in the absence of Cas13 [15,80], causing an RNAi phenomenon. Therefore, individual crRNA and Cas13 controls are necessary to recognize such activity, and further study is required to determine if this can occur in the presence of both Cas13 and crRNA. Another constraint that limits the application of Cas13 is the neurotoxic and embryotoxic effects of some Cas13 enzymes observed in mammalian cells [45,81]. Recently Charles et al. [82] repurposed the inactive Cas13 enzymes for targeted translational repression in bacteria and mitigating their toxicity. Nonetheless, alternative studies are needed to determine the potential of this new system to reduce the toxic effects of Cas13 in different organisms.

## 6. Conclusions

RNA targeting and editing in plants using CRISPR/Cas13 systems can provide great insights into plant transcriptomic studies. CRISPR/Cas13 application in plants has mainly been to impart tolerance against plant viruses. However, it is also emerging as a prominent tool to target mRNA, circRNA, and other non-coding RNAs. In addition, nucleic acid detection using CRISPR/Cas13 is gaining attention in many recent studies for its high specificity, especially in viral diagnostics. Though Cas13 has a few limitations such as a PFS requirement and non-specific collateral activity affecting its editing efficiency, orthologs such as LwaCas13a, PspCas13b, RfxCas13d can be used to avoid the PFS preference, and collateral RNA cleavage of Cas13 has not been observed in plants yet. Therefore, based on current applications and future perspectives, CRISPR/Cas13 systems can potentially emerge into a robust RNA targeting platform in plants, providing novel opportunities in modern agricultural applications.

## Figures and Tables

**Table 2:** Summary table of CRISPR/Cas13 applications in plants.

Application Scope	Cas13 Type	Plant Species	Target	Reference
Viral RNA interference	LshCas13a	*Nicotiana benthamiana*	*Turnip Mosaic Virus* (TuMV)	[40]
		*Arabidopsis thaliana*		[41]
		*Solanum tuberosum*	*Potato Virus Y* (PVY)	[42]
		*Nicotiana benthamiana, Oryza sativa*	*Southern Rice Black-Streaked Dwarf Virus* (SRBSDV), *Rice Stripe Mosaic Virus* (RSMV)	[43]
	LshCas13a, LwaCas13a, PspCas13b, BzCas13b, RfxCas13d	*Nicotiana benthamiana*	*Turnip Mosaic Virus* (TuMV), *Tobacco Mosaic Virus* (TMV), *Potato Virus X* (PVX)	[30]
	LshCas13a, LwaCas13a, PspCas13b, RfxCas13d	*Nicotiana benthamiana, Ipomoea batatas*	*Turnip Mosaic Virus* (TuMV), *Cucumber Mosaic Virus* (CMV), *Sweet Potato Chlorotic Stunt Virus* (SPCSV)-RNase3	[44]
mRNA knockdown	LwaCas13a	*Oryza sativa* ssp. Japonica var. Nipponbare (Protoplasts)	5-enolpyruvylshikimate-3-phosphate synthase (EPSPS), hydroxycinnamoyl transferase (HCT), and phytoene desaturase (PDS)	[25]
	LbaCas13a, LbuCas13a	*Nicotiana benthamiana, Arabidopsis thaliana, Solanum lycopersicum*	PDS transcript	[15]
RNA detection	LwaCas13a, PsmCas13b	(Glyphosate resistant) *Glycine max*	EPSPS from *Agrobacterium* sp. strain CP4 (CP4 EPSPS)	[32]

## Data Availability

Not applicable.
